# Intronic gRNAs for the Construction of Minimal Gene Drive Systems

**DOI:** 10.3389/fbioe.2022.857460

**Published:** 2022-05-12

**Authors:** Alexander Nash, Paolo Capriotti, Astrid Hoermann, Phillipos Aris Papathanos, Nikolai Windbichler

**Affiliations:** ^1^ Department of Life Sciences, Imperial College London, London, United Kingdom; ^2^ Department of Entomology, Robert H. Smith Faculty of Agriculture, Food and Environment, Hebrew University of Jerusalem, Rehovot, Israel

**Keywords:** gene drives, genetic control, *Drosophila*, synthetic biology, genetics

## Abstract

Gene drives are promising tools for the genetic control of insect vector or pest populations. CRISPR-based gene drives are generally highly complex synthetic constructs consisting of multiple transgenes and their respective regulatory elements. This complicates the generation of new gene drives and the testing of the behavior of their constituent functional modules. Here, we explored the minimal genetic components needed to constitute autonomous gene drives in *Drosophila melanogaster*. We first designed intronic gRNAs that can be located directly within coding transgene sequences and tested their functions in cell lines. We then integrated a Cas9 open reading frame hosting such an intronic gRNA within the *Drosophila rcd-1r* locus that drives the expression in the male and female germlines. We showed that upon removal of the fluorescent transformation marker, the *rcd-1r* ^*d*^ allele supports efficient gene drive. We assessed the propensity of this driver, designed to be neutral with regards to fitness and host gene function, to propagate in caged fly populations. Because of their simplicity, such integral gene drives could enable the modularization of drive and effector functions. We also discussed the possible biosafety implications of minimal and possibly recoded gene drives.

## Introduction

Synthetic gene drives are designed to be transmitted to the progeny at super-Mendelian (>50%) frequencies. CRISPR–Cas9-based gene-drive systems have recently been shown to propagate efficiently in laboratory populations of several insects (Raban et al., 2020; Bier, 2022). They are thus seen as novel tools to modify wild populations of organisms, offering new strategies to reduce the impact of vector-borne diseases and eliminate populations of agricultural pests or target invasive species. Arthropod-borne diseases remain at the forefront of gene drive research. They are endemic in more than 100 countries and affect approximately half of the world’s population (Hill et al., 2005). Mosquitoes are the vectors of several diseases of major global public health importance, including malaria and dengue fever. Although the use of currently available malaria control tools over the past 2 decades has greatly reduced malaria cases and deaths, progress has decreased in recent years (WHO, 2021), and eliminating malaria will likely require new development technologies and tools. The dengue virus is a potential threat to an estimated 2.5 billion people, and the last half-century has witnessed a 30-fold increase in the global incidence of dengue (Harapan et al., 2020). Gene drive technology has reached a stage where constructs are capable of reliably eliminating caged mosquito populations (Hammond et al., 2016; Kyrou et al., 2018; Simoni et al., 2020; Hammond et al., 2021a; Hammond et al., 2021b). Gene drives for population replacement have also advanced, and proof of principle has been achieved in mosquito vector species (Gantz et al., 2015; Pham et al., 2019; Adolfi et al., 2020; Carballar-Lejarazu et al., 2020). In addition to these advances, several innovative gene drive designs have been explored in model organisms focused on reducing the rise of resistance or to curtail gene drive spread (Champer et al., 2018; Oberhofer et al., 2019; Champer et al., 2020a; Champer et al., 2020b; Oberhofer et al., 2020; Price et al., 2020).

One approach of interest is the modularization of gene drive functions into a split drive or autonomous/non-autonomous gene drive components which can have advantages and which does not, in itself, reduce the level of the drive (Champer et al., 2019; Nash et al., 2019; Edgington et al., 2020; Kandul et al., 2020; Terradas et al., 2021). It allows for testing individual components of a gene drive strategy, for example, antimalarial effector molecules that require evaluation at a scale large enough so that the detection of expected or unexpected entomological and epidemiological effects is possible before they are widely applied (James et al., 2018). Modularization could allow to safely test such molecules at an ever larger scale moving safely and in defined steps from the laboratory to the field without a strict requirement for geographical isolation. For example, inundative releases of a non-autonomous effector-carrying strain would allow the assay of mosquito fitness and performance under field conditions and detection of any unintended effects prior to deployment. When tested in the absence of an active gene drive element, a non-autonomous effector will not convert the field population, permitting its safe testing (Nash et al., 2019). The release of the same non-autonomous effector strain in combination with a non-driving source of Cas9 could trigger a limited and localized spread of the effector trait and allow the evaluation of its drive performance and perhaps its epidemiological effect. At a later stage, full gene drive of the same effector traits could be enabled by introducing autonomous Cas9 drive elements. Modular gene drives could also be simpler, and each component is designed to feature only the minimal set of genetic modifications needed to be introduced into individual genomes to only those components necessary for global health, agriculture, or ecology. Along these lines, we have been developing a gene drive approach termed as the integral gene drive (IGD), which involves minimal genetic modifications of host genes and a separation of transmission blocking and gene drive functions into separate loci and strains (Nash et al., 2019). We have already shown that effector molecules can be expressed within endogenous mosquito loci and that those can be mobilized into non-autonomous gene drives when a source of Cas9 is provided in trans (Hoermann et al., 2021).

In the current study, we sought to design and test the other necessary component of such a system, i.e., an autonomous integral gene drive capable of expressing Cas9 and a gRNA using the *Drosophila* model. Different design criteria apply to this strategy which requires the modification of germline loci and the hosting of the substantial Cas9 coding sequence within such a locus. The sole purpose of such an autonomous drive construct is to propagate and seed Cas9 within a population while ideally having a minimal impact on the fitness or fertility of the target organism. The latter requires that the gRNA be expressed efficiently from within the same locus and does not interfere with either Cas9 or the host gene function. To achieve this, we designed intronic gRNA modules that could be located within coding sequences of other genes without interfering in their functioning and tested them in *Drosophila* S2 cells. One successful design was then further evaluated in transgenic flies and on the population level.

## Results

### Establishing a Testing Platform for Intron-Encoded gRNAs

**FIGURE 1:**
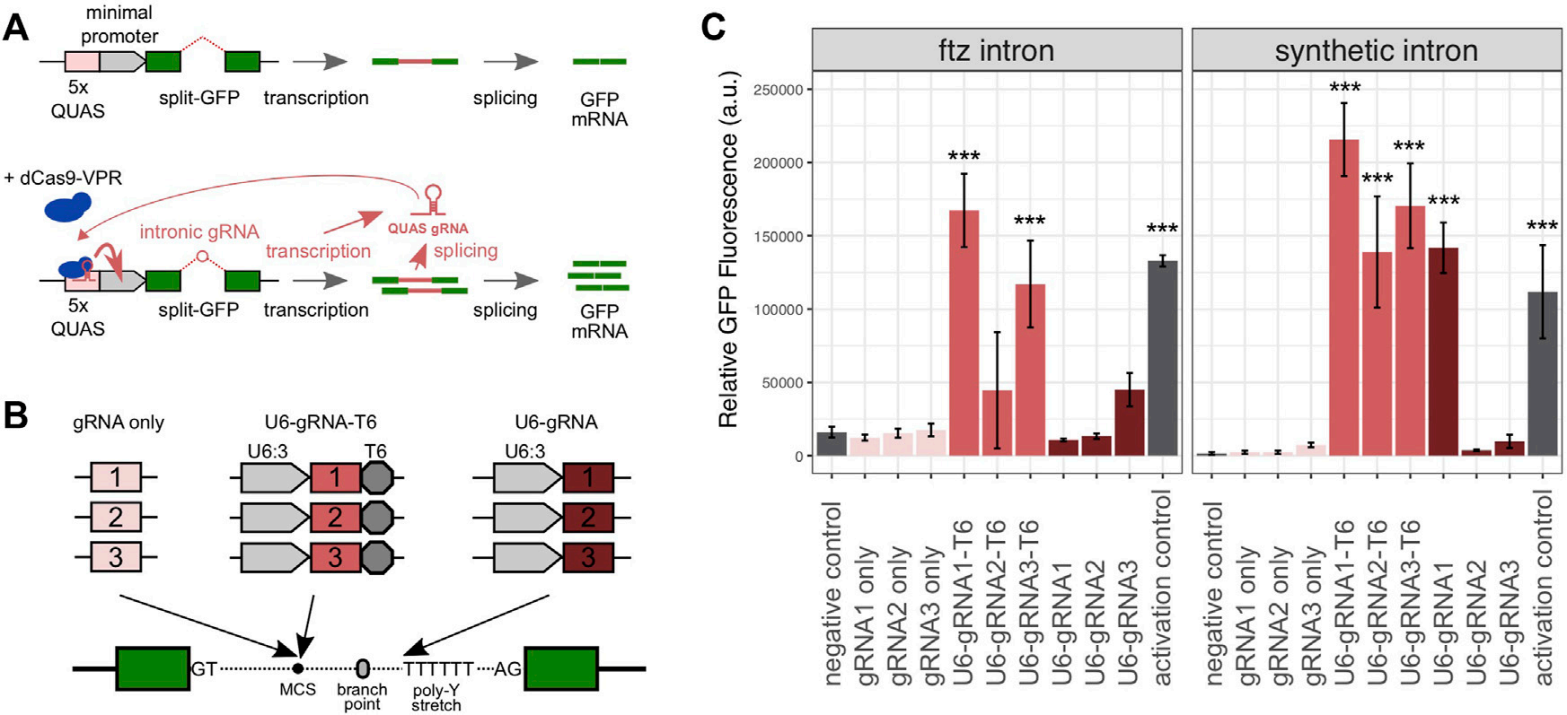
Characterization of intron-encoded gRNAs in *Drosophila* S2 cells. **(A)** Assay consists of a GFP reporter split by the presence of an intron and the dCas9-VPR activator component. In the absence of a gRNA guiding the activator, the reporter exhibits low levels of eGFP fluorescence. Following the activation by the dCas9:gRNA complex at the 5′ QUAS motif, eGFP fluorescence is amplified. This requires both successful splicing and the gRNA expression from within the intron. Relative fluorescence is subsequently measured by flow cytometry, allowing each gRNA configuration to be compared to a negative control (no gRNA) and a positive control (gRNA provided separately). **(B)** Three intronic gRNA designs were tested with or without a U6 promoter, each with three previously characterized QUAS-targeting gRNA spacers, cloned at the multiple cloning site (MCS) near the intronic branch point, or directly adjacent to the polypyrimidine stretch toward the 3′ end of each intron. **(C)** Relative mean eGFP fluorescence and SD from triplicate transfections of each intronic gRNA variant, using either the *ftz* or minimal introns. The *p* values were calculated using one-way ANOVA and Tukey multiple comparisons of means (*p < 0.05; **p < 0.01; and ***p < 0.001).

We first designed a synthetic gene circuit where the successful GFP expression was coupled to the activity of an intron-encoded gRNA (Figure 1A). Specifically, this circuit was designed so that both the splicing of the gRNA-containing intron (located within the reporter gene) and the efficient expression of the gRNA cassette itself were necessary to achieve high levels of eGFP expressions. For this purpose, we used a set of three gRNAs that had previously been described to be capable of recruiting a dCas9 transcriptional activator to the ×5 QUAS sequence (Lin et al., 2015). In this plasmid-based system, we provided the dCas9-VPR-T2A-mCherry activator together with a reporter plasmid containing the 5xQUAS motif upstream of a minimal Hsp70 promoter and the eGFP cassette which in turn hosted the intron constructs to be tested. We assessed two intron variants based on either the *Drosophila melanogaster ftz* intron or a synthetic intron previously characterized in a mammalian cell culture (Kiani et al., 2014). The QUAS-targeting gRNAs were incorporated into the introns upstream of the branch point, as summarized in Figure 1B. We either included the full U6:3 promoter and a T6 terminator or simply the gRNA sequence itself without any additional regulatory sequences. In the former case, we expected that the gRNA expression would be driven independently from the expression of the GFP gene whereas in the latter case, the spliced intronic RNA would itself act as a gRNA, as has been shown before (Figure 1B). A third variant was also generated where the gRNA was located directly on the 5′ strand of the poly-pyrimidine stretch where the leading T nucleotides were meant to act to terminate transcription.

### Testing the Performance of Intron-Encoded gRNAs in *Drosophila* S2 Cells

The reporter and activation constructs were co-transfected into *Drosophila melanogaster* S2 cells to evaluate the performance of gRNA constructs and to simultaneously enable splicing and the transactivation of the eGFP expression. GFP fluorescence was quantified to determine the relative efficacy of each intron-encoded gRNA variant. As a negative control, we employed the *ftz* and synthetic intron base constructs prior to the insertion of any gRNA cassette. As a positive/activation control, we used these base constructs together with the dCas9-VPR activator into which we cloned a cassette co-expressing transactivating gRNA1. As expected, we detected, for both intron variants, low levels of the eGFP expression in the negative controls (Figure 1C). The positive control yielded a strong fluorescent signal relative to the negative control (8.3-fold and 71.3-fold induction in the *ftz* and synthetic constructs, respectively), suggesting that transactivation was successful and that the intron control constructs lacking gRNAs were splicing efficiently. When analyzing combinations of gRNAs and the two intron variants, we found that no significant fluorescent induction was triggered by the constructs containing each gRNA alone (Figure 1C). This suggested that either splicing or the level of gRNAs liberated as spliced RNA was insufficient in these constructs. When we supplied gRNA1 for transactivation by co-transfecting the activation control plasmid, the *ftz* and synthetic minimal constructs carrying gRNA1 did yield a fluorescent signal comparable to that of the respective U6-gRNA1-T6 constructs (Supplementary Figure S1). This suggested that the minimal constructs did not express sufficient gRNA for sufficient transactivation but could splice efficiently when the gRNA was provided in trans. The constructs featuring the U6 promoter and terminator sequences consistently performed best in this assay for both intron types. The U6-gRNA1-T6 construct yielded a fluorescent signal 10.4-fold (*ftz* intron) and 137.5-fold (synthetic intron) higher than the controls. The U6-gRNA1-T6 construct was also the only construct performing significantly better than the activation control (1.9-fold and *p* < 0.0001), although several constructs yielded an overall higher mean level of fluorescence. Only a single experiment (U6-gRNA1 in conjunction with the synthetic intron) featuring gRNA-encoded upstream of the poly-pyrimidine stretch showed a significant level of induction (Figure 1C). Overall, we concluded that full gRNA expression cassettes can be located within small intron sequences, allowing both efficient gRNA expressions and splicing, and we took these designs forward for evaluation in transgenic flies.

### Generation of Integral Gene Drive Strains

**FIGURE 2:**
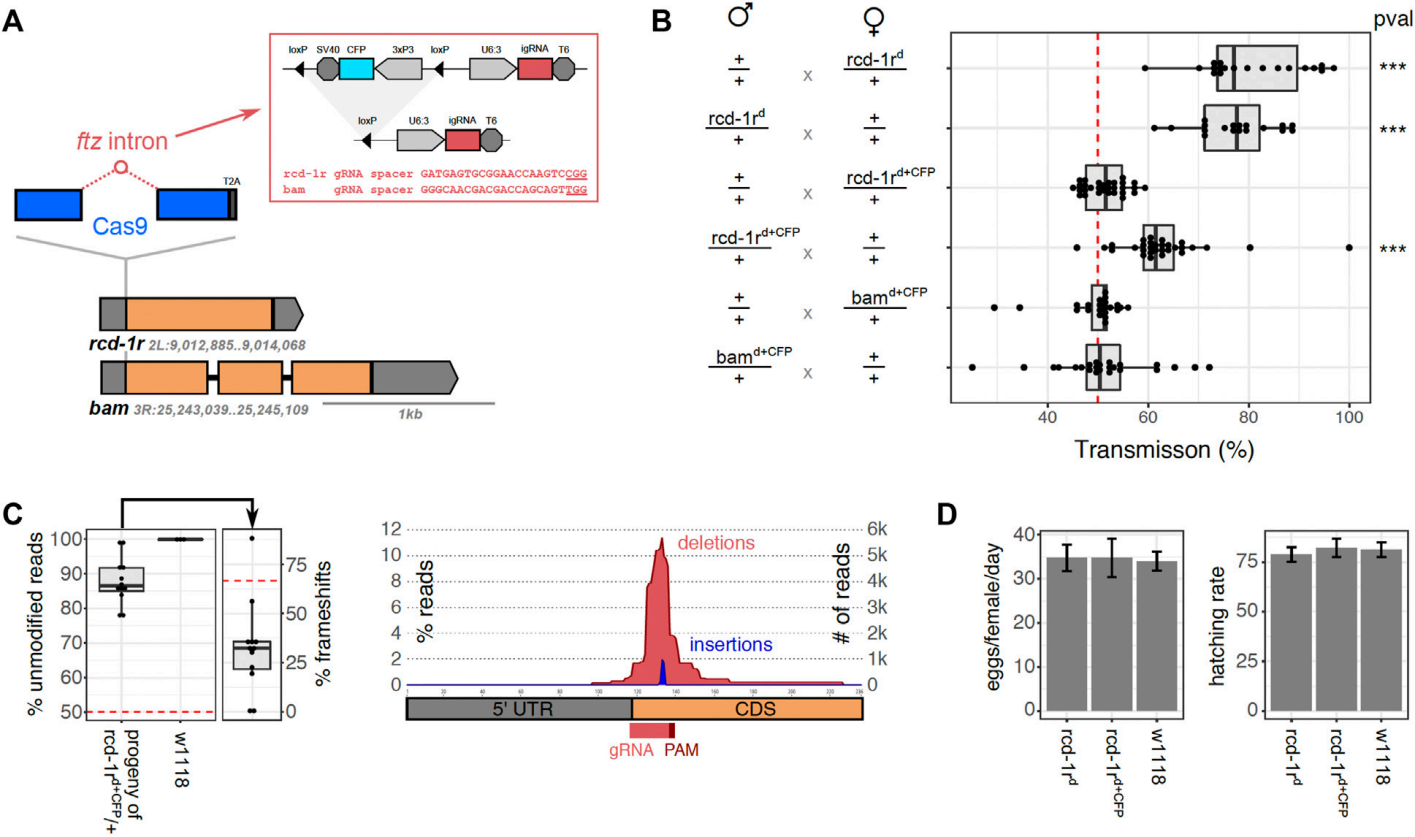
Characterization of integral gene drive traits in transgenic flies. **(A)** Schematic representation showing the design and integration strategy of the Cas9 coding sequence (blue), hosting the *ftz* intron (red) which in turn contains the gRNA expression cassette and the excisable CFP marker cassette, at the endogenous loci *rcd-1r* and *bam* (orange) to which it is translationally linked (T2A). The gRNA target sequences (red) or the gRNA module (red circle) is indicated including the protospacer adjacent motif (underline). **(B)** Homing assays determining the rate of inheritance of the transgene to the progeny of male or female hemizygote flies. Inheritance was calculated as the percentage of the fluorescent progeny (*rcd-1r* ^*d+CFP*^, *bam* ^*d+CFP*^) or by molecular genotyping (*rcd-1r* ^*d*^). The *p* values were calculated using one-way ANOVA and Tukey multiple comparisons of means (*p < 0.05; **p < 0.01; and ***p < 0.001). We performed a minimum of 18 independent crosses per condition. **(C)** Analysis of *rcd-1r* target site amplicons from the pooled progeny of *rcd-1r* ^*d+CFP*^ hemizygote males (n = 12) and a *w1118* wild-type control (n = 3). The left panel shows the median number of modified reads and the number of target site variants that are predicted to cause a frameshift. The right panel shows the size distribution of modified positions around the gRNA target site. **(D)** Analysis of fertility and fecundity of gene drive flies. The left panel shows an analysis of oviposition for homozygous transgenic strains *rcd-1rd + CFP*, *rcd-1r* ^*d*^ and control flies performed in at least 10 independent crosses. The mean and the standard error of the mean for the number of eggs laid per female, per day (left panel), as well as the rate of eggs hatching to become adults (right panel) are shown. No statistically significant difference was found amongst the strains using one-way ANOVA and Tukey multiple comparisons of means (*p < 0.05; **p < 0.01; and ***p < 0.001).

For the generation of autonomous integral gene drives, we chose two *Drosophila melanogaster* target loci, *bam* and *rcd-1r*, the regulatory regions of which had previously been utilized for the successful generation of gene drives in the fly (Chan et al., 2011; Chan et al., 2013; Simoni et al., 2014). These two genes were also chosen because in both cases, we were able to identify Cas9-targetable sites and PAM motif close to the start codons that would allow the insertion of the Cas9 coding sequence upstream of the coding sequence of these genes (Figure 2A). *Bam* (CG10422) has been associated with the fusome, a germ cell–specific organelle, and it contributes to the fate determination of germline stem cells in males and females with loss of function, leading to the production of aberrant sperm and eggs. The *bam* promoter has been frequently used to drive a germline-specific expression in *D. melanogaster*. *Rcd-1r* (CG9573) is a retrogene (Bai et al., 2007) that has been implicated in male fertility with loss-of-function mutants found to be semi-sterile or sterile (Chen et al., 2012). It shows strong expressions in adult testes, and although *rcd-1r* 5′UTR has been assayed mainly for inducing male-specific gene drives, *rcd-1r* itself is also expressed during early embryogenesis, specifically in zygotic germ cell nuclei starting from stage 8 and in early developing gonads (Tomancak et al., 2002; Lecuyer et al., 2007; Tomancak et al., 2007; Hammonds et al., 2013; Wilk et al., 2016). We next designed integration constructs where intron-encoded gRNAs targeting *bam* or *rcd-1r* were located within the Cas9 open reading frame which was designed to link to the host locus *via* a T2A signal (Figure 2A). We chose the *ftz* intron which is derived from *Drosophila*, and the full U6:3-gRNA-T6 design had previously shown good performance in the cell assay. The constructs also featured a removable 3xP3-CFP transformation marker flanked by loxP sites and 5′ and 3′ regions of homology to the *bam* or *rcd-1r* loci. In both cases, the transgenes were successfully integrated into these two germline loci, which were confirmed by PCR genotyping of G1 individuals which were outcrossed to balancer strains. Following intercrosses, we observed that homozygous *bam* ^d+CFP^ male and female individuals were sterile, whereas we managed to successfully establish a homozygous strain *rcd-1r* ^d+CFP^ which showed no obvious fertility defects. In order to remove the 1.6 kb transformation marker cassette flanked by loxP sites which we expected to interfere with correct splicing and host gene expression, we next crossed homozygous *rcd-1r* ^d+CFP^ or TM6B-balanced bam^d+CFP^ heterozygotes to a Cre-recombinase expression strain. The transhemizygote progeny was intercrossed, and their CFP-negative progeny was then propagated in single crosses, and their offspring in turn was screened molecularly for successful excision of the fluorescent marker cassette. Following this strategy, we successfully established a homozygous strain *rcd-1r* ^d^. In the case of *bam* insertion, however, we did not recover any offspring from 10 independent single crosses of markerless individuals. This suggests that although the transgene (now consisting of only the Cas9 coding sequence and the intron-encoded gRNA) was designed to not interfere with the function of the host gene, the presence of the insert did not allow for the sufficient or correct expression of *bam* and was causing sterility.

### Assaying Super-Mendelian Inheritance of the Transgenic Strains

To investigate the gene drive of the constructs established at the two loci, we conducted a series of homing assays (Figure 2B). We crossed male or virgin female *rcd-1r* ^*d*^ hemizygotes, *rcd-1r* ^d+CFP^ hemizygotes, and *bam* ^d+CFP^ hemizygotes with wild-type (*w* ^*1118*^) flies and determined the transmission of the transgene in the progeny. This was either carried out by tracking the fluorescent CFP marker or, in the case of the markerless *rcd-1r* ^*d*^ insertion, by making use of molecular genotyping. Between 18 and 30, single crosses of one male and three females were established for each condition, and crosses that failed to produce offspring were discarded. Figure 2B summarizes the results of these experiments. We found no significant deviation from Mendelian transmission rates for the *bam* ^d+CFP^ transgene (51.3 and 49.2% transgenic progeny for male and female crosses, respectively). This suggests that Cas9 and/or the gRNA were not correctly expressed in these constructs in all likelihood due to the presence of the CFP marker. Given these results and due to the observed sterility of homozygotes, for the remaining experiments, we focused on the *rcd-1r* insertions alone. We did observe a significant gene drive in *rcd-1r* ^d+CFP^ male (62.8% transgenics, *p* < 0.001) but not in the female (51.4%) cross. High rates of transmission were observed for the *rcd-1r* ^*d*^ construct from both hemizygous males (77.1%, *p* < 0.001) and females (80.5%, *p* < 0.001). We concluded that although some levels of the expression of Cas9 occur in the presence of the CFP marker gene in *rcd-1r* ^*d*^ males, more substantial rates of homing were enabled only once the marker cassette had been removed. The rcd-1r gene had previously been used for a male-specific gene drive; here however, we observed the drive in both sexes. Together with the established expression pattern of *rcd-1r* in early embryogenesis, this suggested that the observed gene drive could also be zygotic. To distinguish between the gene drive in the germline and zygotic gene drive, we crossed homozygous *rcd-1r* ^*d*^ */rcd-1r* ^*d*^ males or females with the wild type. In the progeny, we did not find any evidence of somatic homozygosity in 92 genotyped individuals, all of which were *rcd-1r* ^*d*^ */+* heterozygotes, suggesting thus that the *rcd-1r* ^*d*^ gene drive is confined to the germline.

### Analysis of Target Site Variants

In order to examine target site modifications in individuals who failed to inherit the drive construct, wild-type progenies were taken from the *rcd-1r* ^*d,CFP*^ homing cross in pools of 50 individuals. Because we outcrossed transgenic males with wild-type females, this experiment allowed us to understand the variety of indels formed in the absence of selection to retain *rcd-1r* function. Wild-type sized alleles were amplified from a subset of these crosses (n = 12) and compared against pools of *w* ^*1118*^ individuals that represent the pre-existing variation at the *rcd-1r* locus in the laboratory colony (n = 3). Resulting reads were analyzed using CRISPResso2 (Clement et al., 2019), and the indel formation rate was determined (Figure 2C). Reads were pooled across all biological replicates and analyzed for frequency of substitutions, deletions, and insertions proximal to the *rcd-1r* target site. We found no strong evidence of pre-existing target site variations at the *rdc-1r* locus in the *w* ^*1118*^ background, but 12.1% of reads from the homing cross were found to carry indels. We found that the majority of indels were predicted to affect the rcd1-r coding sequence, but interestingly, less than the predicted 2/3 of indel events were predicted to result in a translational frameshift (32.4%). We attributed this discrepancy to the presence of a common indel variant found in 7/12 replicates and representing a total of 11% of all detected variant reads. It is likely generated by an ATGAGT microhomology with a predicted MMHEJ score of 240.8 (Bae et al., 2014) and results in a 24-bp deletion which, although it maintains the *rcd-1r* reading frame, leads to the elimination of the original start codon and its context (Supplementary Table S1).

### Analysis of *rcd-1r* ^*d*^ Fertility

In order to determine whether insertion of the IGD drive component affected the fitness of the transgenic *rcd-1r* strains, phenotypic assays were performed to measure basic life history traits related to fertility and fecundity. We quantified the egg output and subsequent hatching rates of homozygous *rcd-1r* ^*d*^ and *rcd-1r* ^*d*,CFP^ strains and the *w* ^*1118*^ controls. Individual homozygote females and males were crossed in vials and allowed to mate for 24 h. At least eight replicates were performed per strain, with assays performed over 72 h, flipping mated females into a fresh vial every 24 h. The egg output was quantified per female, per day for each transgenic strain (Figure 2D). The vials were maintained at 25°C in order to quantify the rate of eclosion. Three days after the eclosion of pupae started, the number of adults in each vial was counted, and the hatching rate was calculated (Figure 2D). No statistically significant difference was observed between the *rcd-1r* ^*d*^ and *rcd-1r* ^*d*,CFP^ drive lines, when compared to a *w* ^*1118*^ control line in terms of fertility or hatching rate. Given that *rcd-1r* had previously been implicated in male fertility, this suggested that at this locus, the IDG components, even in the presence of the full fluorescent marker cassette, did not interfere with fly fertility.

### Population Cage Experiments

**FIGURE 3:**
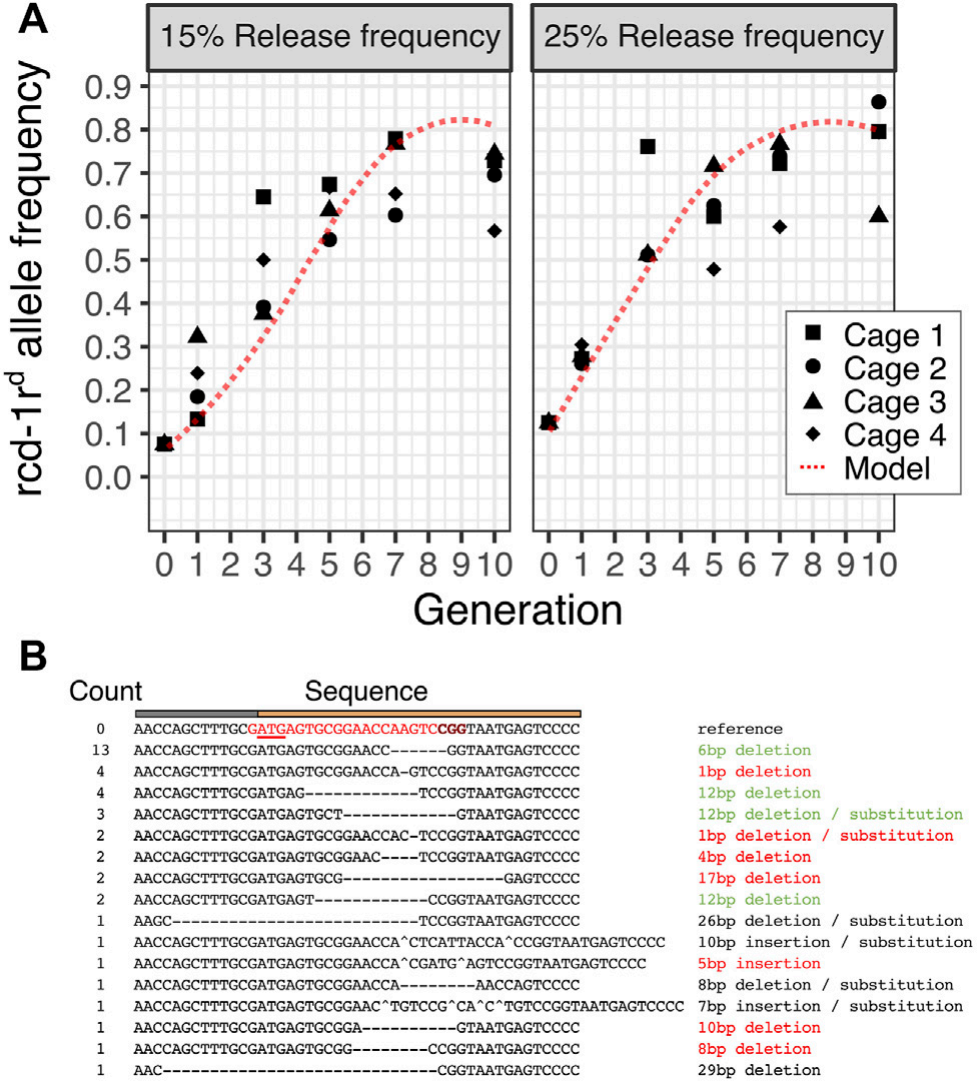
Population dynamics of the *rcd-1r* ^*d*^ gene drive. **(A)** The predicted allele frequency of *rcd-1r* ^*d*^ in four replicate cages maintained for 10 generations and sampled at generations 1,3,5,7, and 10 at two initial release frequencies (15% or 25% hemizygote *rcd-1r* ^*d*^ corresponds to a starting allele frequency of 7.5% or 12.5%, respectively) is shown. The dotted red line represents the predicted frequency according to the mean of 25 runs of a stochastic model of the *rcd-1r* ^*d*^ drive. A minimum of 31 and a mean of 42 individuals were genotyped per condition, cage, and generation. **(B)**. Summary of target site variants identified by Sanger sequencing in generation 10 using hemizygous individuals from all eight cage populations. The putative impact of the mutation as disruptive (red), possibly tolerable (green), and ambiguous/unclear (black) is indicated.

We next performed population cage experiments to determine the dynamic behavior of the *rcd-1r* ^*d*^ integral gene drive in caged *Drosophila* populations of 200 individuals over 10 fly generations. We performed this experiment in four replicate populations and two release conditions where hemizygous *rcd-1r* ^*d*^ males represented 15% or 25% of the starting male populations. This corresponds to 7.5 and 12.5% starting allele frequencies of the *rcd-1r* ^*d*^ drive allele, respectively. Because *rcd-1r* ^*d*^ is a markerless gene drive, we used molecular genotyping to determine the presence of hemizygous and homozygous transgenic individuals at generations 1, 3, 5, 7, and 10. We compared the observed dynamic to that predicted by a stochastic agent-based model which was parameterized with the fitness and homing rates for *rcd-1r* ^*d*^. We found that as predicted by the model which assumes that maintaining the *rcd-1r* function is necessary for male but not female fertility, the *rcd-1r* ^*d*^ drive spreads rapidly but reaches a maximum allele frequency of approximately 80% with a similar peak frequency under both release conditions. These frequencies are slightly less than those predicted by the models and could be due to the effects that we did not account for computationally, for example, low levels of maternal Cas9 deposition into the embryo. This experiment validates the results of the individual genetic crosses and shows a successful drive of a minimal integral gene drive at the population level. We performed Sanger sequencing of non-transgenic individuals to determine target site variants that had accumulated in these cage populations by generation 10. Out of a total of 40 samples, 13 carried a 6-bp deletion which was detected in six out of the eight cage populations (Figure 3B, Supplementary Table S1). This suggests that selection for maintaining the *rcd-1r* function leads to the rise of a prominent variant featuring a two-amino acid deletion at the protein’s N-terminus.

## Discussion

Here, we sought to generate an autonomous gene drive consisting of a minimum number of genetic components in *Drosophila melanogaster*. Our aim was to design a neutral gene drive that would seed a population with a source of Cas9 but cause no other intended phenotypes. We first characterized, in cell culture experiments, intron modules that allowed both intron splicing and the U6-driven expression of the intron-encoded gRNAs. Although promoterless intron variants were also tested and the liberation of spliced introns as gRNAs had previously shown to be attainable, such variants failed to achieve high levels of activity in our assay. We next showed that U6-driven gRNA-expressing introns, when embedded within the Cas9 open reading frame, could constitute a functional gene drive in transgenic flies. Such a module, when integrated at the N-terminus of the germline-expressed *rcd-1r* gene and translationally linked to its expression, showed high levels of the gene drive in the male and female germline, while at the same time we could not detect any negative effects on fly fertility. This approach was not as successful at the *bam* locus where the introduced sequences evidently interfered with the host gene expression, leading to sterility. The intronic gRNAs we described could have other applications besides the gene drive. For example, intronic gRNAs could be integrated at or around rare polymorphisms of genes of interest as they would be neutral with regards to the host gene function. If such gRNAs target the wild-type allele, they could, when paired with a source of Cas9, drive the allelic conversion of a tissue or a population. Such a strategy could be used to study the function of rare alleles.

We studied the behavior of the *rcd-1r* gene drive at the population level and found that the spread of *rcd-1r* ^*d*^ matched the dynamic predicted by a simple discrete-generation model. The model assumes that the loss of the *rcd-1r* function (R2 homozygote genotype) leads to a 95% reduction in male fertility, and thus, these R1 alleles will be selected. When we analyzed the generation of resistant alleles, we found that in single crosses, in the absence of selection, there was a preferred repair outcome which was likely the result of microhomology around the *rcd-1r* gRNA target site. This variant, however, was not detected at all in the cage populations where the most common variant was found to carry a two-codon deletion, which was likely selected for its ability to maintain *rcd-1r* function. Markerless gene drives, as described herein, can only be detected by molecular methods. When the target site and genomic location of the gene drive is unknown and when, as we show, there is a dearth of additional exogenous sequence elements, genotyping would likely require targeting the Cas9 ORF itself. Due to the possibility of recoding of the ORF (or the use of alternative CRISPR endonucleases) and the presence of introns, it is possible that the standard detection method could easily be circumvented. Although we used a conventional Cas9 ORF, the presence of the gRNA intron would cause standard Cas9 primers to not yield the correct product for the *rcd-1r* ^*d*^ gene drive. In similar designs, only whole genome sequencing or protein-based detection methods could reliably identify gene drive individuals. These considerations should inform discussions around the biosecurity of the gene drive as it is possible to design minimal and recoded gene drives that could propagate in populations undetected (e.g., if a deliberate or accidental release of an unregulated or “garage” gene drive were to occur). Our results thus demonstrated that the generation of autonomous gene drives with a minimum number of genetic components is achievable (consisting of only a Cas9 coding sequence hosting an intronic gRNA). Further experiments, including experiments in non-model target organisms such as malaria mosquitoes, will be required to see if this approach is more broadly applicable. In particular, the targeting of genes with a more conserved N-terminus and males and females could avoid some of the issues encountered at the *rcd-1r* locus. Targeting highly conserved sequences has been shown to alleviate some of the problems related to the formation of resistant alleles (Kyrou et al., 2018); however, the N-terminal may offer fewer such conserved targetable sites. We have already used the methods described herein to develop analogous non-autonomous gene drives in the malaria mosquito (Hoermann et al., 2021). These traits expressed antimalarial effectors from within mosquito host genes and carried gRNA-expressing introns based on the designs we described here. When combined with Cas9 expressor strains, autonomous and non-autonomous gene drives could constitute a modular system to test gene drives and their effector mechanisms at an increasing scale moving from the laboratory to the field.

## Materials and Methods

### Cell Culture and Transfection

Experiments were performed in Schneider 2 cells (Thermo Fisher Scientific, United Kingdom). Cells were cultured at 25°C in an ambient atmosphere. Cells were maintained in a complete growth medium composed from 90% Schneider’s *Drosophila* medium (Thermo Fisher Scientific, United Kingdom), and 10% FBS (Sigma-Aldrich, United Kingdom). Cells were maintained in T-25 flasks, with 0.2-μM filter caps. Visualization was performed using a Nikon TMS inverted microscope and a ×10 confocal lens. Cells were passaged biweekly, at 1:4 ratios, at which point they had achieved a density of >1 × 10^6^ cells/ml. The cell count was performed using a Scepter 2.0 Cell Counter. Confirmation of the mycoplasma-free status was performed by PCR. Constructs used for experimental work in S2 cells were prepared for transfection by being midiprepped using *E. coli* TOP10 cells. Following transformation, individual colonies were selected and used to inoculate 5 ml of LB-Miller starter cultures, supplemented with ampicillin. Starter cultures were incubated for 8 h at 37°C in a shaking incubator at 220 rpm. They were then used to inoculate 500-ml falcon flasks containing 100 ml of LB-Miller broth, supplemented with ampicillin. Falcon flasks were incubated overnight at 37°C in a shaking incubator at 220 rpm.

### DNA Constructs

Annotated DNA plasmid sequences have been provided in Supplementary Material S1. Briefly, sequences for the constructs used for the cell-based assay were derived from pQUAST (Addgene plasmid #24349) by the insertion of gene-synthesized fragments and validated by Sanger sequencing. To generate U6:3-gRNA-T6 gRNA cassettes for the generation of gene drive constructs, spacers (*bam GTC​GGG​CAA​CGA​CGA​CCA​GCA​GT*, *AAA​CAC​TGC​TGG​TCG​TCG​TTG​CCC*; and *rcd-1r GTC​GAT​GAG​TGC​GGA​ACC​AAG​TC*, *AAA​CGA​CTT​GGT​TCC​GCA​CTC​AT*) were synthesized as two complementary ssDNA oligos (Eurofins Genomics, Ebersberg, Germany), with BbsI compatible overhang sites in the pCFD3-dU6:3gRNA (Addgene plasmid #49410) (Port et al., 2014), which were then cloned into the Bsp119I site within pBS-Hsp70-SpCas9-ftz, followed by the subsequent insertion of the 3xP3:CFP:SV40 cassette. For each locus, 1-kb homology arms were synthesized (ATUM, CA, United States), and BbsI or BaeI sites were introduced to produce a linearized backbone into which the drive cassette was inserted by the Gibson assembly.

### Flow Cytometry

Fluorescence in S2 cells was analyzed by using a three-laser BD LSRFortessa analyzer. Live, single cells were selected by gating forward and side scatter. For each condition analyzed, at least 500 gated events were recorded, with three replicates per condition. The following voltages were used—forward scatter (FSC): 170 V, side scatter (SSC): 253 V, 530-nm channel: 390 V, and 610-nm channel: 591 V. Excitation of eGFP was performed using a 488-nm blue laser and by using a 530/30 bandpass filter. The excitation of mCherry was performed using a 561-nm yellow laser and by using a 610/20 bandpass filter. Transfection of single color constructs was used for the construction of compensation matrices to account for spectral overlap.

### Transgenic Flies

Embryos were dechorionated in 50% bleach, prior to microinjection and aligned on 10 × 10 mm coverslips. The needles were pulled using a P1000 Sutter micropipette puller (Sutter, United Kingdom). Injections were conducted with *w* ^*1118*^ embryos at 500 ng/μl, consisting of a 400 ng/μl drive construct and 100 ng/μl pnos-Cas9 helper plasmid. Following microinjection, embryos were retained on coverslips and placed onto 100 mm × 100 mm agar plates. Upon hatching, first instar larvae were placed into a yeast paste and transferred to plugged vials to mature at 22°C. All stocks were maintained at 18°C, while all experiments were conducted at 25°C under Arthropod Containment Guidelines Level 2 conditions.

### Cre-Mediated Reporter Excision

*Rcd-1r* drive lines were generated by performing 10 single crosses of *rcd-1r* ^d,CFP^ with *y1 w* ^*67c23*^; *sna* ^*Sco*^/*CyO*, and Pw [+mC] = CrewDH1 virgins to excise the 3xP3:CFP:SV40 reporter cassette located within the Cas9-located *ftz* intron of the drive component. G1 progenies were all markerless and were intercrossed to produce homozygous *rcd-1r* ^*d*^ G2 progenies. Excision of the CFP reporter cassette was confirmed by Sanger sequencing.

### Fertility Assay

The oviposition and hatching assay (Simoni et al., 2014) was performed using the *w* ^*1118*^ line as a control. Virgin females and males were collected and aged for 24 h. Single crosses were subsequently performed, with mating being allowed to proceed for 48 h. Males were subsequently removed, and females were allowed to lay eggs in vials containing standard yeast food for three consecutive days, with flies transferred into a new vial every 24 h. At least eight replicates were performed per line, with the number of eggs per female per day quantified. The number of eclosed adults arising from said eggs was then quantified, with at least eight replicates observed per line.

### Homing Assay

Hemizygotes were obtained by crossing single *rcd-1r* and *rcd-1r* ^d,CFP^ males to *w* ^*1118*^ virgins. In the case of *bam* balanced *w* ^*1118*^, *bam*/TM6B and Tb [1] males were crossed with *w* ^*1118*^ virgins. Subsequent G1 heterozygotic progenies were screened such that only *w* ^*1118*^ *bam* individuals were selected to act as parents for homing assay crosses. Hemizygous progenies were collected such that the females were virgins, and all individuals were 1–3 days old. Single males were crossed with three females in each cross. Male and female hemizygous transgenics were assayed separately to determine their sex-specific effects. In cases where replicates failed to yield offspring, they were discarded and where lines carried a CFP reporter cassette, the gene drive carrying the progeny was identified using an Olympus MVX10 fluorescent microscope for fluorescence. A markerless *rcd-1r* progeny was genotyped by extracting genomic DNA using Chelex 100 resin. An extraction solution was produced by suspending 1.25 g of Chelex resin in 100 ml of RNase-free water supplemented with 4 ml of proteinase K solution (20 mg/ml). This solution was added in 100 μl aliquots to each well containing a fly. Plates were sealed using adhesive plate foils. Incubation occurred in an Eppendorf Thermomixer-C, for 2 h at 55°C and 700 rpm, and then, proteinase K was inactivated by incubation for 20 min at 99°C. Plates were stored at −20°C. PCR reactions using primers TAG​CAA​AGT​CAG​GGC​GTA​GC and CAC​CGG​GAT​AAG​CCC​ATC​AG using REDTaq DNA polymerase were conducted as follows: 98°C for 2 min, followed by 35 cycles of 98°C denaturation step for 20 s, a 59°C annealing step for 30 s, and a 72°C extension step for 30 s, followed by a final 72°C extension step for 7 min, and then a 4°C hold. PCR reactions were visualized using 1% agarose gels, and the presence of a 609-bp band was considered to be a positive indication of a transgenic individual.

### Amplicon Sequencing

Amplicon sequencing was performed on a CFP-negative progeny of the *rcd-1r* ^d,CFP^ homing assay. In total, 50 CFP-negative G2 progenies were selected at random from a given biological replicate, and pooled genomic DNA extraction was performed for 12 replicates. Then, 200 control *w* ^*1118*^ flies of a commensurate age were selected, and pooled genomic DNA extraction was performed in three replicates. Qubit quantified yields were normalized to 50 ng/μl using Tris-EDTA. PCR was then performed using primers ACA​CTC​TTT​CCC​TAC​ACG​ACG​CTC​TTC​CGA​TCT​GAC​GCT​TTT​CCA​CAG​CAT​GG and GAC​TGG​AGT​TCA​GAC​GTG​TGC​TCT​TCC​GAT​CTT​TCG​GTC​CTT​TCT​CGC​TTG​A producing an amplicon of or around 320 bp. Primers included a partial scaffold compatible with Illumina NGS sequencing. PCR conditions were as follows: 98°C denaturation step for 2 min, followed by 23 cycles of 98°C for 10 s, 57°C for 10 s, and 72°C for 30 s, followed by a final 72°C extension for 7 min, and a 4°C hold. Successful amplification of PCR products was confirmed by loading 5 μl of each sample onto a 1% agarose gel, running at 100 V for 60 min, and visualizing. PCR products were subsequently purified using a QIAGEN MinElute PCR Purification kit (QIAGEN, CA, United States), and purified DNA products were eluted in 10 μl Tris-EDTA. Purified DNA was quantified using a Qubit fluorometer as before and normalized to 20 ng/μl, in at least 20 μl of the total volume using Tris-EDTA. Samples were processed by Genewiz (Genewiz, Leipzig, Germany), and the data were processed using CRISPResso2 (Clement et al., 2019).

### Population Cage Experiments

Experiments were performed in a 250-ml conical bottle, plugged with cellulose acetate plugs (VWR, United Kingdom) and filled with 50 ml of standard yeast-based fly food (12lt water, 240 gr polenta, 90 gr agar, 960 gr fructose, 1,200 gr brewer’s yeast, 60 ml Nipagin 15% w/v in ethanol, and 90 ml propionic acid). Flies were allowed to mate for 5 days, before being recovered using CO_2_, and stored at −80°C for subsequent analysis. Three days after G1 individuals had eclosed, the adults were retrieved from each bottle and counted. Then, 200 individuals were selected at random and placed into a fresh bottle to begin the next generation. Any excess flies were frozen at −80°C for later analysis. This cycle was repeated until generation 10. To genotype DNA, extraction was performed as described, and multiplex PCR reactions were setup in 96-well plates, corresponding to each gDNA plate. Primers AGA​AGC​TGT​CGT​CCA​CCT​TG, ACG​TGC​TTT​CGG​TCC​TTT​CT, and AGG​TGT​TCT​TGC​TCA​GCT​CC that bind to the transgene and the endogenous locus generate a product of 586 bp when the *rcd-1r* ^*d*^ allele is present and a 292-bp product for the wild-type allele. We used the REDTaq DNA polymerase mastermix (VWR, United Kingdom) as follows: denaturation at 98°C for 2 min, followed by 35 cycles of the 98°C denaturation step for 20 s, 59°C annealing step for 30 s, and the 72°C extension step for 40 s, followed by a final 72°C extension step for 7 min, and then a 4°C hold. Plates were sealed, as before, with an adhesive foil. A total of 10 *rcd-1r* ^*d*^ hemizygous G10 samples from each replica of both release conditions were further analyzed through Sanger sequencing in order to determine the presence of target site variants.

### Gene Drive Model

We employed a simple object-oriented stochastic agent-based discrete generation model written in C#. The model allowed to fully account for the genetic interactions of autonomous and non-autonomous CRISPR-based drive elements and the generation and tracking of R1 and R2 resistance alleles at all modeled loci. Briefly, we considered populations of 200 individuals where WT, R1, R2, and drive constituted the possible genotypes at the *rcd-1r* locus. We included the experimentally observed drive parameters and a 95% loss of male fertility for a loss of the *rcd-1r* function (R2 homozygotes). In each generation, all adults were randomly mated, and 200 offspring were randomly selected for constituting the next generation.

## Data Availability

The original contributions presented in the study are included in the article/Supplementary Material, further inquiries can be directed to the corresponding author.

## Supplementary Material

Supplementary Figure S1 Testing intronic gRNA designs in the presence of the activation control to determine splicing levels. Relative mean eGFP fluorescence and SD from triplicate transfections of each intronic gRNA variant, using either *ftz* or minimal introns. The *p* values were calculated using one-way ANOVA and Tukey multiple comparisons of means (**p*<0.05; ***p*<0.01; and ****p*<0.001).
