# Supplementary figures and images for: Intronic gRNAs for the Construction of Minimal Gene Drive Systems
Source: Front Bioeng Biotechnol. 2022 May 12;10:857460. doi: 10.3389/fbioe.2022.857460 (PMC9133698; doi:10.3389/fbioe.2022.857460)

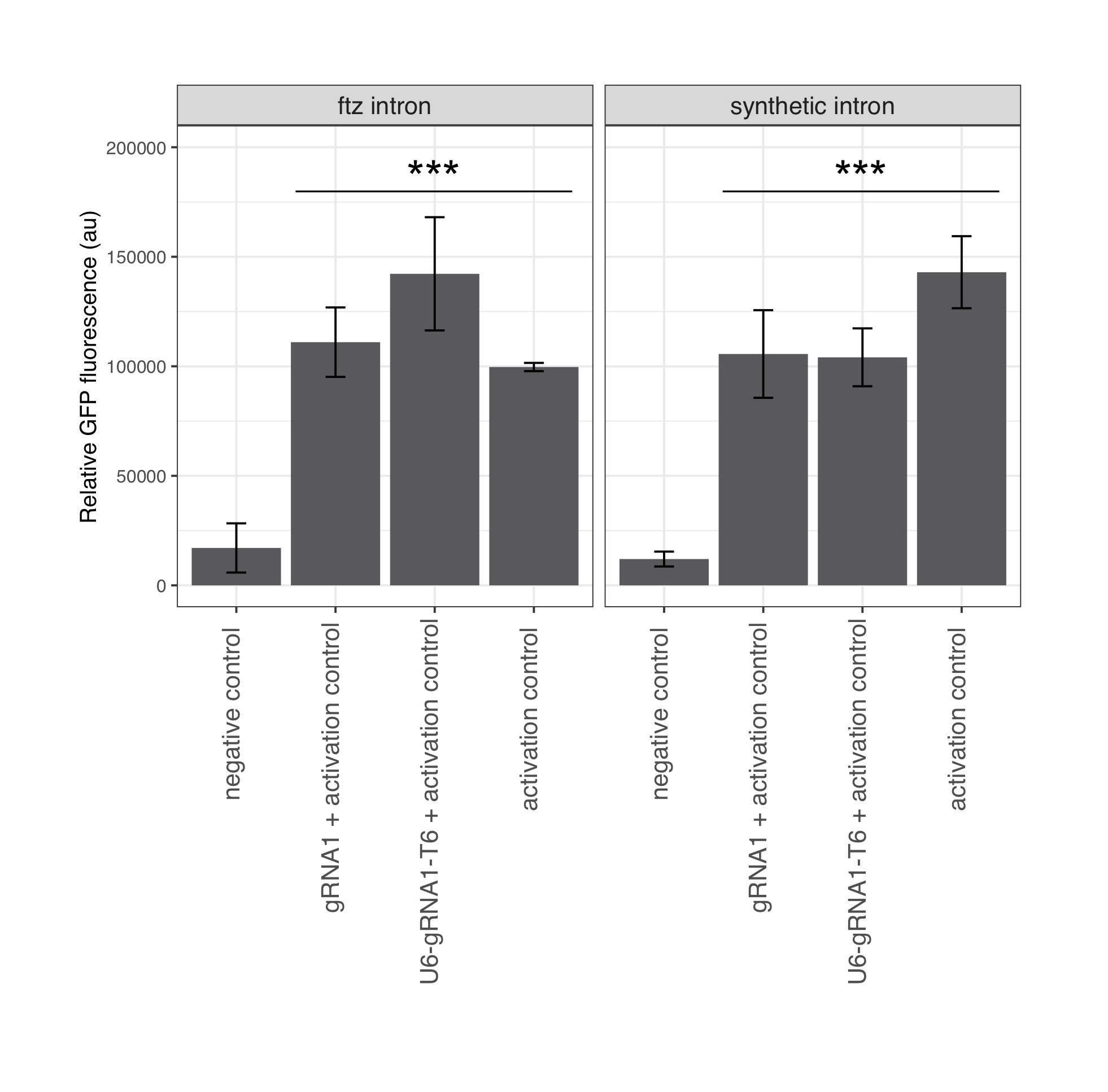

Supplement: Supplementary file 1 [file Image1.JPEG]
